# Derivation of Induced Trophoblast Cell Lines in Cattle by Doxycycline-Inducible piggyBac Vectors

**DOI:** 10.1371/journal.pone.0167550

**Published:** 2016-12-01

**Authors:** Takamasa Kawaguchi, Dooseon Cho, Masafumi Hayashi, Tomoyuki Tsukiyama, Koji Kimura, Shuichi Matsuyama, Naojiro Minami, Masayasu Yamada, Hiroshi Imai

**Affiliations:** 1 Laboratory of Reproductive Biology, Graduate School of Agriculture, Kyoto University, Kyoto, Japan; 2 Research Center for Animal Life Science, Shiga University of Medical Science, Shiga, Japan; 3 Animal Feeding and Management Research Division, NARO Institute of Livestock and Grassland Science, Tochigi, Japan; Michigan State University, UNITED STATES

## Abstract

Trophectoderm lineage specification is one of the earliest differentiation events in mammalian development. The trophoblast lineage, which is derived from the trophectoderm, mediates implantation and placental formation. However, the processes involved in trophoblastic differentiation and placental formation in cattle remain unclear due to interspecies differences when compared with other model systems and the small repertoire of available trophoblast cell lines. Here, we describe the generation of trophoblast cell lines (biTBCs) from bovine amnion-derived cells (bADCs) using an induced pluripotent stem cell technique. bADCs were introduced with piggyBac vectors containing doxycycline (Dox)-inducible transcription factors (*Oct3⁄4(POU5F1)*, *Sox2*, *Klf4*, and *c-Myc*). Colonies that appeared showed a flattened epithelial-like morphology similar to cobblestones, had a more definite cell boundary between cells, and frequently formed balloon-like spheroids similar to trophoblastic vesicles (TVs). biTBCs were propagated for over 60 passages and expressed trophoblast-related (*CDX2*, *ELF5*, *ERRβ*, and *IFN-τ*) and pluripotency-related genes (endogenous *OCT3/4*, *SOX2*, *KLF4*, and *c-MYC*). Furthermore, when biTBCs were induced to differentiate by removing Dox from culture, they formed binucleate cells and began to express pregnancy-related genes (*PL*, *PRP1*, and *PAG1*). This is the first report demonstrating that the induction of pluripotency in bovine amniotic cells allows the generation of trophoblastic cell lines that possess trophoblast stem cell-like characteristics and have the potential to differentiate into the extra-embryonic cell lineage. These cell lines can be a new cell source as a model for studying trophoblast cell lineages and implantation processes in cattle.

## Introduction

Mammalian blastocysts are composed of two distinct cell types: the inner cell mass and the trophectoderm. The trophectoderm is the first cell type that differentiates from pre-implantation embryos at the blastocyst stage. The trophectoderm cell lineage plays an important role in implantation and placental formation [1]. However, the processes trophoblastic differentiation and placental formation in cattle are poorly understood due to interspecies differences compared with mice and humans. In cattle, a blastocyst hatches in the uterus at day 9 of pregnancy and initiates the rapid elongation of the trophectoderm at around day 12 [2]. The extraembryonic membrane extends throughout the entire uterine horns by day 24 and subsequently attaches to endometrial cells [3]. At the beginning of implantation and throughout pregnancy, trophoblastic binucleate cells are differentiated from mononuclear cells [4]. Binucleate cells are fused with uterine epithelial cells and formed trinucleate cells and these cells play similar roles to syncytiotrophoblasts [5]. During this peri-implantation period, trophoblastic cells produce a number of molecules such as interferon-tau (IFN-τ), placental lactogen (PL), prolactin-related proteins (PRPs) and pregnancy-associated glycoproteins (PAGs). IFN-τ is a cytokine that mononuclear trophoblast cells of ruminant conceptuses secrete [2, 6–8], and the signals support the recognition of pregnancy [6, 9]. IFN-τ is detected on days 7–8, increases on day 14, peaks on days 19–20 and declines soon after the conceptus attaches to the uterine epithelium [10, 11]. In contrast, PL, PRPs, and PAGs are hormones that are secreted by binucleate cells and play a main role in the fetal-maternal interface [4].

Recently, a decrease in the conception rate of cattle has become a serious problem for many countries. Dunne [12] reported that most embryonic losses in heifers have occurred before day 14 of pregnancy. Given that this period is closely related to the time of elongation of trophoblasts, it is likely that the development and differentiation of trophoblast cells affect conception ability in cattle. In rodents, trophoblast stem cells (TSCs) have been derived from the polar trophectoderm of blastocysts and retain the capacity to differentiate into all trophoblast derivatives of the later placenta *in vitro* [13, 14], and these cells have been used to investigate their role in the placenta [15]. In contrast, authenticated TSCs have not been generated from ungulate species, although primary trophoblast cell lines have been produced from conceptuses from sheep and goat [16], pig [17–19], and cattle [20–22]. Many of these cell lines grow continuously in culture without apparent senescence and display characteristics expressed in trophoblast cells, but they likely represent a differentiation state beyond TSCs in terms of morphology, the presence of binucleate cells in colonies and gene expression related to binucleate cells. Therefore, there are no standard procedures for culturing TSCs in these species until now.

Since the first generation of induced pluripotent stem cells (iPSCs) [23], the technique for inducing pluripotency by ectopic expression of transcription factors in somatic cells has allowed the generation and maintenance of iPSCs in species including cattle [24] in which it has been difficult to isolate and culture embryonic stem cells [25–27]. Recently, the iPS cell technique has also allowed the generation of trophoblast cell lines from somatic cells in pigs [28] and in humans [29]. This cell lineage also showed trophoblast-like characteristics such as an epithelial-type morphology, the expression of trophoblast-related genes and the formation of trophoblastic vesicles (TVs). However, to date, there are no reports regarding the generation of a trophoblast stem cell line in cattle.

In this study, to provide cattle trophoblast stem cell lines, we attempted to establish induced trophoblast cells (biTBCs) from bovine amnion-derived cells (bADCs) and estimate the cellular characteristics and potential to differentiate into the trophoblast cell lineage.

## Materials and Methods

### Ethics statements

All cattle were fed grass silage-based diet *ad libitum*. All procedures involving the care and use of animals were approved by the Animal Research Committee of NARO institute of Livestock and Grassland Science.

### Isolation of bovine amnion-derived cells (bADCs) and fetal liver-derived cells (bFLCs)

A bovine amnion layer was harvested from a female Japanese black cattle fetus after 50 days of gestation at the National Institute of Livestock and Grassland Science, Japan. The amnion was mechanically peeled away from the chorion and allantois, divided into small pieces with fine surgical scissors, and dissociated by incubating for 2 hours at 37°C with 0.3% collagenase (Wako, Osaka, Japan) in Dulbecco’s modified Eagle medium (DMEM, Invitrogen, Carlsbad, CA, USA) containing 10% fetal bovine serum (FBS, JRH Biosciences, Lenexa, KS, USA). After collagenase digestion, the cell suspension was maintained at room temperature for 5 min and then poured through a cell strainer; the filtered suspension was then centrifuged at 200 *g* for 5 min. The precipitated cells were cultured in DMEM containing 10% FBS, penicillin (Sigma-Aldrich, St. Louis, MO, USA), and streptomycin (Sigma-Aldrich). When the cells reached confluence, they were cryopreserved in liquid nitrogen until use.

Bovine liver tissue was isolated from a female Japanese black cattle fetus at 68 days of gestation at the National Institute of Livestock and Grassland Science, Japan. The liver was divided into small pieces with fine surgical scissors, and dissociated by incubating for 2 hours at 37°C with 0.1% collagenase in DMEM. After collagenase digestion, the cell suspension was diluted with DMEM containing 10% FBS and then poured through a cell strainer; the filtered suspension was then centrifuged at 200 *g* for 5 min. The precipitated cells were cultured in DMEM containing 10% FBS, penicillin, streptomycin, and primocin (InvivoGen, San Diego, CA, USA). When the cells reached confluence, they were cryopreserved in liquid nitrogen until use.

### Cell culture

bADCs and bFLCs were maintained on collagen-coated (Nitta Gelatin, Osaka, Japan) dishes in somatic cell medium consisting of DMEM containing 10% FBS, 50 ng/ml epidermal growth factor (EGF, Calbiochem, San Diego, CA, USA), penicillin, and streptomycin. The cells were dissociated enzymatically with TrypLE Select (Invitrogen) for further propagation.

Bovine trophoblast CT-1 cells, a generous gift from Dr. Kazuhiko Imakawa, The University of Tokyo, were propagated as described previously [2]. In this study, CT-1 cells were cultured on plastic plates coated with Matrigel at 38.5°C in air with 5% CO_2_ in DMEM containing 10% FBS) supplemented with 4.5 g/L D-glucose (Invitrogen), nonessential amino acids (Invitrogen), 2 mM glutamine (Invitrogen), 1 mM sodium pyruvate (Invitrogen), 55 μM b-mercaptoethanol (Invitrogen), penicillin, and streptomycin. Hereinafter, the cells were called ‘TB cells’.

biTBCs were generated by introducing Dox-inducible PB vectors into bADCs or bFLCs as described below. biTBCs were first generated in iPS medium consisting of Dulbecco's Modified Eagle Medium/Nutrient Mixture F-12 (DMEM/F12, Invitrogen) containing 20% Knockout Serum Replacement (KSR, Invitrogen), 2 mM L-glutamine (MP Biomedicals, Santa Ana, CA, USA), 1×MEM nonessential amino acids (NEAA, Invitrogen), 0.1 mM 2-mercaptoethanol (2-ME, Wako), penicillin and streptomycin supplemented with 2.0 μg xmL doxycycline (Dox, Sigma-Aldrich) and 5 ng/mL human basic fibroblast growth factor (bFGF, Wako or ReproCELL, Kanagawa, Japan). In subsequent experiments, biTBCs were generated and maintained in trophoblast medium (TBM) consisting of DMEM/F12 containing 10% FBS, 2 mM L-glutamine, 1×NEAA, 0.1 mM 2-ME, penicillin and streptomycin supplemented with 2.0 μg ⁄mL Dox. Culture medium was changed every other day. Cells were subcultured every 7 days by physically splitting the cells into clumps using a pulled Pasteur pipette and maintaining them on 35 mm diameter cell culture dishes (IWAKI, Tokyo, Japan) on a feeder layer of 3–5×10^5^ cells SNL cells [30, 31] inactivated with 10 μg /ml mitomycin C (Sigma-Aldrich).

For feeder-free culture, biTBCs were split into cell clumps, and the clumps were transferred onto collagen-coated dishes. Cells were maintained in TBM and subcultured every 7 days.

All cultures were maintained in a humidified incubator at 38.5°C with 5% CO_2_ in air.

### Generation of biTBCs using Dox-inducible PB vectors

**Fig 1:**
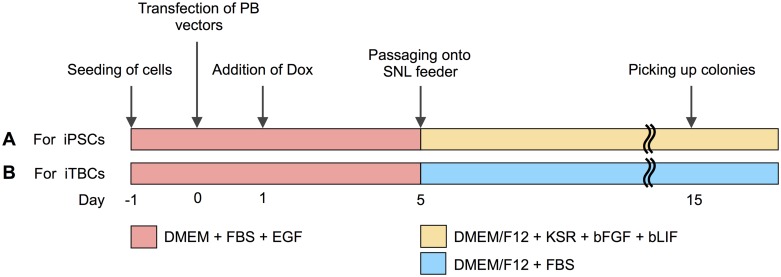
Generation of bovine-induced trophoblastic cells (biTBCs) using Dox-inducible PB vectors. (A) Timeline for the establishment of biTBCs in culture medium used for human iPSCs. (B) Timeline for the establishment of biTBCs in culture medium used for bovine trophoblast cells (TBM).

bADCs were plated at 1.25×10^5^ cells per 35-mm dish in the culture medium without antibiotics and incubated overnight. Cells were then transfected using Lipofectamine LTX (Invitrogen). Briefly, equal amounts (0.4 μg) of hyPBase vectors (pCAG-hyPBase) [32], PB vectors with reprogramming factors (PB-TET-OKS and pPB-TET-c-Myc) [33–35], the rtTA PB vector (PB-CAG-rtTA Adv, Addgene), and/or the TagRFP PB vector (pPBCAG-TagRFP-IH) [35], 2 μL of Plus reagent (Invitrogen) and 10 μL of Lipofectamine LTX transfection reagent were diluted and mixed in 400 μL Opti-MEM medium (Invitrogen). The DNA-lipid complexes were then added to the culture dish. The culture medium was changed 6 hours after transfection. One day after transfection, the culture was supplemented with 2.0 μg ⁄mL Dox. Four days after the Dox addition, cells were dissociated with TrypLE Select, 1×10^5^ cells were reseeded on a SNL feeder layer, and the medium was replaced with iPS medum or TBM. Fourteen days after the addition of Dox, primary colonies were mechanically collected and transferred onto a SNL feeder layer in 4-well plates. The medium was changed every day or every other day, depending on cell growth (Fig 1).

The original vectors including PB-TET-MKOS (a tetracycline inducible polycistronic vector containing transcription factors in the following order: *c-Myc*, *Klf4*, *Oct3/4*, and *Sox2*), PB-CAG-rtTA Adv and pCX-OKS-2A (a polycistronic retroviral vector containing transcription factors in the following order: *Oct3/4*, *Klf4*, and *Sox2*) were obtained from Addgene (plasmids 20959, 20910, and 19771, respectively) [33, 34]. The empty PB vector and the c-Myc PB vector (pPB-TET-c-Myc) were kind gifts from Dr. Hitoshi Niwa at the RIKEN Center for Developmental Biology. The hyPBase vector (pCMV-hyPBase) was a kind gift from Dr. Keisuke Yusa at the Sanger Institute. To generate pCAG-hyPBase, pCMV-hyPBase was inserted into the pCAGGS vector [36].

### Alkaline phosphatase and immunofluorescence assays

Alkaline phosphatase activity in biTBCs was measured using the Vector Alkaline Phosphatase Substrate kit (Vector, Burlingame, CA, USA). For immunofluorescence analysis, cells were fixed with PBS containing 3.7% paraformaldehyde for 10 min at room temperature. After washing with PBS, cells were blocked with 5% bovine serum albumin (Sigma-Aldrich) and 0.1% Triton X-100 (Sigma-Aldrich) for 45 min at room temperature and then incubated overnight at 4°C with primary antibodies directed against OCT3/4 (1:50, SC-9081, Santa Cruz, Dallas, TX, USA), NANOG (1:250, AB5731, Millipore, Billerica, MA, USA), CDX2 (1:100, MU392A-UC, BioGenex, Fremont, CA, USA), or IFN-τ (1:1000, Operon, Tokyo, Japan). Alexa Fluor 488 goat anti-mouse IgG (1:500, Invitrogen) or Alexa Fluor 488 goat anti-rabbit IgG (1:500, Invitrogen) were used as secondary antibodies. Nuclei were stained with 1 μg ⁄mL Hoechst 33342 (Sigma-Aldrich).

### Reverse transcription PCR

Total RNAs of cells were prepared using the TRIzol reagent (Invitrogen). DNase I (Takara, Shiga, Japan) was added to preparations to avoid genomic DNA contamination. For reverse transcription, ReverTra Ace (Toyobo, Osaka, Japan) and Random Primer (Invitrogen) were used. PCR was performed with ExTaq (Takara). PCR reactions were set up as follows: 94°C for 2 min followed by 25–40 amplification cycles (94°C for 20 s, 60°C for 30 s, and 72°C for 30–45 s). The reactions included a final elongation step at 72°C for 7 min. Primer sequences are shown in S1 Table.

### Trophoblastic vesicle formation and differentiation of biTBCs

To assess the differentiation potential of biTBCs, these cells were cultured in low-adhesion culture dishes to induce differentiation similar to the method for inducing embryoid body formation from iPSCs [37]. biTBCs were physically split into cell clumps and transferred to MPC (2-methacryroyloxyethyl phosphorylcholine)-treated round-bottom dishes (Nunc, Roskilde, Denmark) in TBM supplemented with 2.0 μg ⁄mL Dox. After 3 days of culture, the medium was changed to fresh medium without Dox and cultured for another 3 days.

The differentiation potential of biTBCs was further examined by culturing the cells without Dox for a long period. biTBCs cultured in TBM supplemented with Dox were physically split into cell clumps and transferred to collagen-coated dishes in TBM in the absence of Dox for 20 to 30 days. The attached cell colonies were subcultured approximately every 10 days. After the culture, total RNA from cells was collected using the TRIzol reagent, and PCR analysis was performed as described above. Immunofluorescence analyses were also performed as described above.

## Results

### Generation of biTBCs using Dox-inducible piggyBac vectors

**Fig 2:**
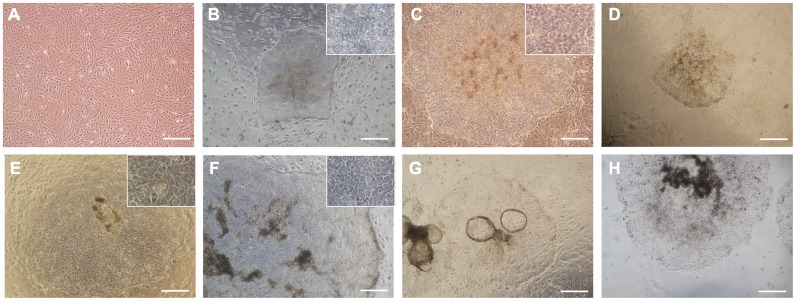
Phase-contrast images of bovine-induced trophoblastic cells (biTBCs). (A) Bovine amniotic cells (bADCs). (B) iPSC-like colonies appearing in the culture medium used for human iPSCs. (C) Trophoblast-like colonies appearing in the culture medium used for human iPSCs. (D) Primary trophoblast-like colonies appearing in the trophoblast cell medium at day 14. (E) Established biTBCs derived from bADCs. (F) Established biTBCs derived from bovine fetal liver cells (bFLCs). (G) Formation of trophoblastic vesicles (TVs) in central areas of colonies during culture. (H) Established biTBCs cultured on collagen-coated dishes. (A)-(H) scale bars = 500 μm.

bADCs isolated from a female Japanese black cattle fetus at 50 days of gestation originally exhibited a heterogeneous population consisting of epithelial and fibroblastic cells in culture (Fig 2A). During trials for the generation of bovine-induced pluripotent stem cells (biPSCs) from bADCs using Dox-inducible PB vectors in culture medium used for human iPSCs, a small portion of the colonies appeared in a similar morphology as human iPSCs (Fig 2B and S1A–S1C Fig). However, approximately 30% (3 out of 10 colonies, 2 out of 9 colonies, and 3 out of 10 colonies at each experiment) of the colonies showed relatively flattened epithelial-like morphology similar to cobblestones and had more definite cell boundaries between cells (Fig 2C). When the transfected cells were cultured in medium used for culturing bovine trophoblast cells (TBM) [21], the cobblestone-like colonies also appeared at day 8, and approximately 70% (6 out of 8 colonies, 4 out of 6 colonies, and 4 out of 6 colonies at each experiment) of the colonies exhibited trophoblast-like morphology at day 14 (Fig 2D and S1D–S1F Fig). The colonies were then mechanically picked up and transferred onto a fresh SNL feeder layer and subcultured (Fig 2E). To examine whether trophoblastic types of colonies can be obtained not only from the extra-embryonic cell lineage but also from the embryonic cell lineage, bovine fetal liver-derived cells (bFLCs) were used. Introduction of Dox-inducible PB vectors into bFLCs also resulted in the appearance of trophoblast-like colonies (Fig 2F), and these colonies were propagated at least 5 passages under the same culture conditions.

Approximately 20 cell lines (biTBCs) were generated from 1×10^5^ transfected bADCs, and two (iTB#1, iTB#2, and iTB#3) were further examined to determine cell characteristics. Both cell lines were subcultured every 7 days and stably propagated for over 60 passages. During the culture of these cell lines, they began to form a balloon-like morphology similar to trophoblastic vesicles (TVs), and some detached into the medium (Fig 2G and S1G Fig). The TVs frequently occupied the central areas of the colonies. At the time of subculture, biTBCs were split into small cell clumps and transferred onto a fresh SNL layer. The cell clumps easily transformed into TVs and approximately half of the cells newly outgrew but the other half remained suspended. Thus, medium changes were performed with care not to remove floating cell clumps.

biTBCs that were cultured on a SNL feeder layer were split into cell clumps, and then the clumps were transferred onto collagen-coated dishes. The cell clumps also formed TVs and produced new extended outgrowths of TVs in a similar manner and morphology as that of biTBCs cultured on a SNL feeder layer (Fig 2H). The cells could be stably passaged on collagen-coated dishes at least 10 times.

## Characterization of biTBCs

**Fig 3:**
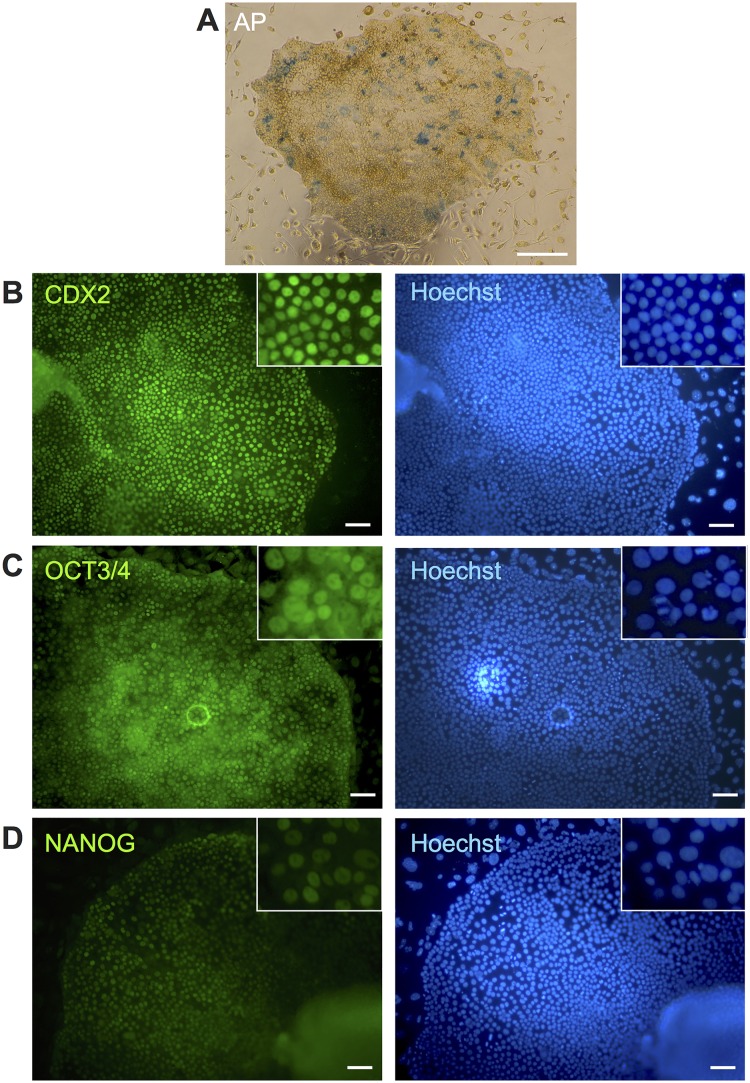
Characterization of bovine-induced trophoblastic cells (biTBCs). (A) Alkaline phosphatase activity in biTBCs. (B) CDX2 expression in biTBCs. (C) OCT3/4 expression in biTBCs. (D) NANOG expression in biTBCs. (A) scale bars = 500 μm. (B)-(D) scale bars = 100 μm.

**Fig 4:**
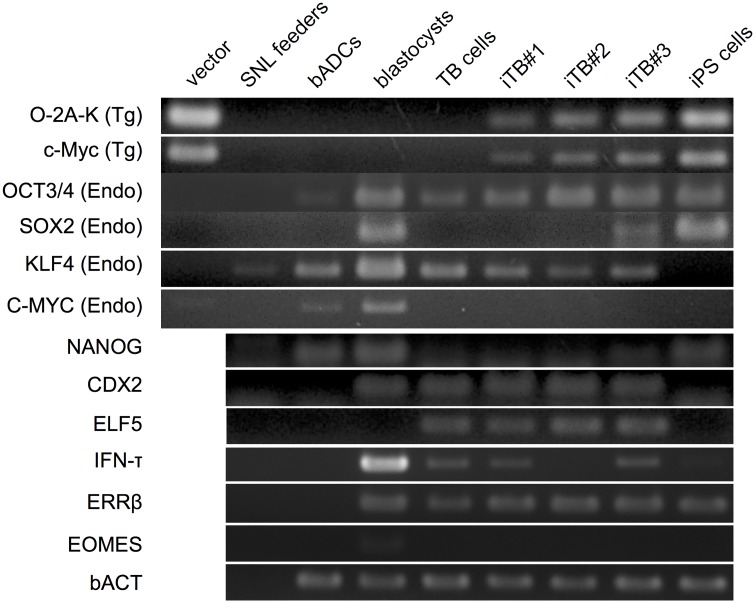
Endogenous and exogenous expression of genes related to pluripotent stem cells or trophoblast cells in bovine-induced trophoblastic cells (biTBCs). mRNA expression was evaluated by reverse-transcription polymerase chain reaction (RT-PCR). vector, plasmid DNA of PB vectors; SNL feeder, SNL feeder cells; bADCs, bovine amnion-derived cells; TB cells, trophoblast cells derived from CT-1 cells; iTB#1, biTBC line #1; iTB#2, biTBC line #2; iTB#3, biTBC line #3; iPS cells, iPS cells derived under the iPS medium condition.

To elucidate the differences between biTBCs and biPSCs, biTBCs were measured for their alkaline phosphatase (AP) activity, a well-known pluripotent marker. The two established biTBC lines were only partially positive for AP activity (Fig 3A and S2A and S2F Fig). To further examine the characteristics of these cells, the expression of pluripotent-related and trophoblast-related genes in biTBCs was assessed. Immunocytochemical analysis showed that the cells expressed the caudal-related homeobox 2 transcription factor (CDX2) required for TSC specification and maintenance and IFN-τ (Fig 3B and S2 and S3 Figs). On the one hand, the cells expressed OCT3/4 (Fig 3C and S2 and S3 Figs) but did not express or only weakly expressed NANOG (Fig 3D and S2 Fig), which are well-known transcription factors required for pluripotent stem cell self-renewal. RT-PCR analysis showed that they expressed trophoblast-related genes including *CDX2*, *ELF5*, *ERRβ*, and *IFN-τ*, although the expression of *IFN-τ* in iTB#2 was relatively low (Fig 4). These cells also expressed pluripotency-related genes including endogenous *OCT3/4*, *SOX2*, *KLF4*, and *c-MYC* (Fig 4). Transgene expression was also detected because the cells were maintained in culture containing Dox (Fig 4).

## Differentiation Potential of biTBCs

**Fig 5:**
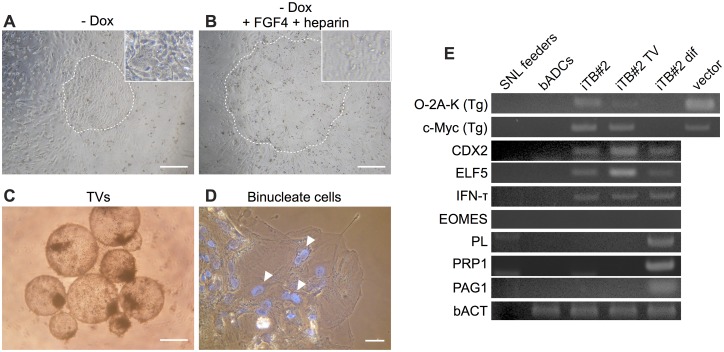
Differentiation potential of bovine-induced trophoblastic cells (biTBCs). (A) Phase-contrast image of biTBCs cultured in the absence of Dox (- Dox) for 7 days. The circled dotted area indicates the colony. (B) Phase-contrast image of biTBCs cultured in the absence of Dox and in the presence of FGF4 and heparin for 7 days. The circled dotted area indicates the colony. (C) Phase-contrast image of TVs generated from biTBCs. (D) Phase-contrast image of differentiated biTBCs. Nuclei were stained with Hoechst. Arrowheads indicate binucleated cells. (E) Endogenous and exogenous gene expression in differentiated biTBCs. iTB#2: Induced trophoblast cell line #2; iTB#2 TV: TVs generated from biTBC line #2 by culturing in low-adhesion culture dishes for 6 days; iTB#2 dif, biTBC line #2 after culturing in the absence of Dox for over 30 days. (A)-(C) scale bars = 500 μm. (D) scale bars = 70 μm.

To examine the effects of Dox on the cellular characteristics of biTBCs, Dox was removed from culture. For 2 passages after culturing in the absence of Dox, the cells readily changed their cobblestone-like morphology into a more epithelial-like appearance (Fig 5A) and lost the ability to form TVs. These observations ware also found even when FGF4 and heparin, which support the derivation and proliferation of murine TSCs [13], were added in culture (Fig 5B).

To assess the differentiation potential of biTBCs, the cells were cultured in low-adhesion culture dishes to induce differentiation similar to the method for inducing embryoid body formation from iPSCs [37] (S1H Fig). Even after the cells were cultured for 6 days, biTBCs did not form embryoid bodies but produced TVs similar to those formed during their culture (Fig 5C). When iTB#2 cells were cultured in the absence of Dox for over 30 days on collagen-coated dishes, they lost their original morphology and some of the differentiated cells formed binucleate cells (Fig 5D). RT-PCR analysis showed that differentiation-induced iTB#2 no longer expressed transgenes but began to express pregnancy-related genes generally expressed in binucleate cells such as *PL*, *PRP1*, and *PAG1* after 30 days of culture in the absence of Dox (Fig 5E).

## Discussion

Trophoblastic cell functions and cell lineages are characterized based on a large number of trophoblast cell lines including TSCs in mice [13, 14]. However, studies regarding trophoblast cell lines in cattle are limited, and the cellular characteristics are poorly understood because of the lack of an *in vitro* culture system for trophoblast cells and their differentiation in culture. Here, we report the generation of new trophoblast cell lines derived from bovine somatic cells by introducing transcription factors used for the generation of iPS cells. Our established trophoblast cell lines (biTBCs) exhibited several criteria of trophoblast stem cells regarding their morphology, cellular behavior, gene expression, and differentiation potential.

After introducing transcription factors by piggyBac vectors, morphologically different colonies from iPS cells appeared even in medium consisting of KSR and FGF2, which are usually used for the generation and maintenance of human iPSCs [38]. These colonies were readily distinguishable from iPSC-like colonies according to their morphology because the colonies exhibited relatively flattened epithelial-like morphology similar to cobblestones and had a more definite cell boundary between the cells. Judging from the morphology, we assumed that the cells were trophoblast cells. Therefore, KSR and FGF2 were replaced by FBS in the culture medium (TBM) because previously reported bovine trophoblast cell lines were usually maintained in FBS-containing medium [21]. As a result, TBM facilitated the emergence of colonies with the trophoblast cell morphology, suggesting that the FBS prompted the induction of stem cell colony differentiation into the trophoblastic cells. Interestingly, trophoblastic cell lines were established not only from amnion-derived cells but also from fetal liver-derived cells, suggesting that biTBCs could be generated not only from extra-embryonic but also from embryonic cell lineages. The timing for the appearance of iPSC-like colonies and that of biTBCs was approximately the same at around day 8. However, it is not clear if the derivation of biTBCs is provided through a transient and metastable iPS cell state or a direct conversion to biTBCs without such an intermediate cell state. Human embryonic stem cells (hESCs) tend to spontaneously convert to trophoblastic cells under standard culture conditions [39] and rapidly differentiate into trophoblast cells upon exposure to BMP4 and related growth factors [40, 41]. Therefore, it is likely that bovine somatic cells underwent cellular reprogramming by the introduction of pluripotency-related transcription factors and readily settled into a trophoblastic cell state.

During the culture of biTBCs, cells frequently formed TVs. This phenomenon is also observed in trophoblast cell lines previously reported in cattle [21]. In addition, when biTBCs were transferred and maintained on collagen-coated dishes, they could also stably propagate for over 10 passages, indicating that our generated biTBCs have similar cellular characteristics in culture as previously established trophoblast cell lines from bovine embryos [21].

biTBCs exhibited partial AP activity, which is known as a pluripotent stem cell marker. This activity has also been observed in embryo-derived TSCs in mice and rats and TBCs generated from porcine somatic cells through the introduction of transcription factors (iTR cells) [28]. Thus, a portion of the population of trophoblast cell lines maintains AP activity; however, the significance of this cell population is obscure in the trophoblastic cell lineage. Moreover, RT-PCR showed that these cells express trophoblast-specific genes, such as *ELF5*, *CDX2*, and *SOX2*, which were also expressed in mouse TSCs [42, 43]. In addition, biTBCs express *IFN-τ*, an antiluteolytic factor responsible for preventing the regression of the maternal corpus luteum and supporting the sustainability of pregnancy for cattle uteri [7, 44]. IFN-τ together with CDX2 is thought to be expressed from mononuclear trophoblast cells or undifferentiated trophoblast cells in ruminants [8, 44]. These results indicate that biTBCs have a heterogeneous population of trophoblast cells and possibly possess cellular characteristics such as those of trophoblast stem cells.

biTBCs expressed endogenous *OCT3/4*, a well-known pluripotent marker. In mouse pluripotent stem cells, OCT3/4 antagonizes CDX2 function and reciprocally regulates their expression, and OCT3/4 expression decreases as trophoblasts differentiate [45–47]. However, co-expression of OCT3/4 and CDX2 has been reported in the trophectoderm of bovine blastocysts, and OCT3/4 expression continues in the trophectoderm (TE) of late blastocysts although CDX2 is still expressed in this tissue [48]. The endogenous CDX2 expression in the TE does not cause a decrease in OCT4 expression, suggesting that bovine OCT4 expression is not simply repressed by CDX2 at the late blastocyst stage [48]. These species-specific differences in *OCT3/4* expression between mice and cattle have been caused by a deficiency in AP2 (TFAP2) binding sites, which are regulatory regions of the OCT3/4 promoter [49]. These repressive sequences should be unique in mouse OCT3/4 regulatory regions and absent in other species including human, rabbit, and pig [48]. In fact, *OCT3/4* expression in the TE at the blastocyst stage is not downregulated in these species [50–52]. Moreover, the species differences in the relationship between OCT3/4 and CDX2 can be attributed to the difficulty in generating biTBCs even by controlling the ectopic expression of transcription factors in the presence of Dox.

When the established biTBCs were cultured in the absence of Dox, they readily changed their morphology and lost the ability to form TVs even in the presence of FGF4 and heparin in culture. A potential solution for achieving the cultivation of iTBCs in the absence of Dox may be to optimize the culture conditions. However, even in the case of mouse TSCs in culture, culture conditions remain complex and require feeder cells or conditioned medium and fetal bovine serum (FBS), fibroblast growth factor 4 (FGF4) and heparin [20–22]. Recently, more defined culture conditions for the derivation and maintenance of murine TSCs have been reported [42] in which TSCs proliferate well with their stem cell characteristics and differentiation ability in media in the absence of FBS supplemented with insulin, transferrin, and low levels of the cytokines FGF4 and TGF-ß1. In addition, a number of fibroblast growth factors such as FGF1, FGF2, and FGF10 are shown to be involved in development of bovine embryos and expression of IFN-τ in cattle [53–55]. This study provides a model for screening for optimal culture conditions for generating authentic bovine TSCs.

biTBCs failed to form EBs after they were cultured in low adhesive culture dishes, but they alternatively formed TVs. Furthermore, when biTBCs were induced to differentiate by culturing in the absence of Dox for over 30 days, they formed binucleate cells and began to express pregnancy-related genes such as PL1, PAG1, and PRP1. The formation of binucleate cells differentiated from mononuclear cells is a characteristic of trophoblast cells in cattle [44, 56], and PL1, PAG1 and PRP1 are genes expressed from binucleate cells [8, 44]. Therefore, the biTBCs in this study have the potential to be differentiated into later stages of the trophoblast cell lineage.

In conclusion, this study reports for the first time that the induction of pluripotency in bovine cells allows for the generation of trophoblastic cells that have trophoblast stem cell-like characteristics and the potential to become differentiated into an extra-embryonic cell lineage. The established cell lines can be a new cell source as a model for studying the trophoblast cell lineage and implantation processes in cattle.

## Supporting Information

S1 Fig Phase-contrast images. (A)–(C) Appearance of colonies at day 8 (A), day 11 (B), day 14 (C) in iPS medium. (D)–(F) Appearance of colonies at day 8 (D), day 11 (E), day 14 (F) in TB medium. (G) TB cells derivated from CT-1 cells. (H) EBs from iPS cells. (A)–(H), scale bars = 500 μm.
(TIF)

S2 Fig Characterization of TB cells and bADCs. (A) Alkaline phosphatase activity in TBcells. (B) OCT3/4 expression in TB cells. (C) NANOG expression in TB cells. (D) IFN-τ expression in TB cells. (E) CDX2 expression in TB cells. (F) Alkaline phosphatase activity in bADCs. (G) OCT3/4 expression in bADCs. (H) NANOG expression in bADCs. (I) IFN-τ expression in bADCs. (J) CDX2 expression in bADCs. (A), (F) scale bars = 500 μm. (B)–(E), (G)–(J), scale bars = 100 μm.
(TIF)

S3 Fig Characterization of biTBCs and biPSCs. (A) IFN-τ expression in biTBCs. (B) CDX2 (red) and OCT3/4 (green) expression in biTBCs. (C) IFN-τ expression in biPSCs. (D) CDX2 expression in biPSCs. (A)-(D) scale bars = 100 μm.
(TIF)

S1 Table Primer sequences. (XLSX)

## References

[1] Pfeffer PL, Pearton DJ. Trophoblast development. Reproduction. 2012;143(3):231–46. 10.1530/REP-11-0374 22223687

[2] Sakurai T, Bai H, Bai R, Arai M, Iwazawa M, Zhang J, et al Coculture system that mimics in vivo attachment processes in bovine trophoblast cells. Biol Reprod. 2012;87(3):60 10.1095/biolreprod.112.100180 22723465

[3] Degrelle SA, Campion E, Cabau C, Piumi F, Reinaud P, Richard C, et al Molecular evidence for a critical period in mural trophoblast development in bovine blastocysts. Dev Biol. 2005;288(2):448–60. 10.1016/j.ydbio.2005.09.043 16289134

[4] Shimada A, Nakano H, Takahashi T, Imai K, Hashizume K. Isolation and characterization of a bovine blastocyst-derived trophoblastic cell line, BT-1: development of a culture system in the absence of feeder cell. Placenta. 2001;22(7):652–62. 10.1053/plac.2001.0702 11504534

[5] Hoffman LH, Wooding FB. Giant and binucleate trophoblast cells of mammals. J Exp Zool. 1993;266(6):559–77. 10.1002/jez.1402660607 8371098

[6] Roberts RM, Cross JC, Leaman DW. Interferons as hormones of pregnancy. Endocr Rev. 1992;13(3):432–52. 10.1210/edrv-13-3-432 1385108

[7] Kimura K, Spate LD, Green MP, Murphy CN, Seidel GE, Roberts RM. Sexual dimorphism in interferon-tau production by in vivo-derived bovine embryos. Mol Reprod Dev. 2004;67(2):193–9. 10.1002/mrd.10389 14694435

[8] Suzuki Y, Koshi K, Imai K, Takahashi T, Kizaki K, Hashizume K. Bone morphogenetic protein 4 accelerates the establishment of bovine trophoblastic cell lines. Reproduction. 2011;142(5):733–43. 10.1530/REP-11-0275 21862694

[9] Imakawa K, Anthony RV, Kazemi M, Marotti KR, Polites HG, Roberts RM. Interferon-like sequence of ovine trophoblast protein secreted by embryonic trophectoderm. Nature. 1987;330(6146):377–9. 10.1038/330377a0 2446135

[10] Guillomot M, Michel C, Gaye P, Charlier N, Trojan J, Martal J. Cellular localization of an embryonic interferon, ovine trophoblastin and its mRNA in sheep embryos during early pregnancy. Biol Cell. 1990;68(3):205–11. 1695857 10.1016/0248-4900(90)90309-q

[11] Imakawa K, Kim MS, Matsuda-Minehata F, Ishida S, Iizuka M, Suzuki M, et al Regulation of the ovine interferon-tau gene by a blastocyst-specific transcription factor, Cdx2. Mol Reprod Dev. 2006;73(5):559–67. 10.1002/mrd.20457 16489630

[12] Dunne LD, Diskin MG, Sreenan JM. Embryo and foetal loss in beef heifers between day 14 of gestation and full term. Anim Reprod Sci. 2000;58(1–2):39–44. 10700643 10.1016/s0378-4320(99)00088-3

[13] Tanaka S, Kunath T, Hadjantonakis AK, Nagy A, Rossant J. Promotion of trophoblast stem cell proliferation by FGF4. Science. 1998;282(5396):2072–5. 9851926 10.1126/science.282.5396.2072

[14] Roberts RM, Fisher SJ. Trophoblast stem cells. Biol Reprod. 2011;84(3):412–21. 10.1095/biolreprod.110.088724 21106963 PMC3043125

[15] Sahgal N, Canham LN, Canham B, Soares MJ. Rcho-1 trophoblast stem cells: a model system for studying trophoblast cell differentiation. Methods Mol Med. 2006;121:159–78. 16251742

[16] Miyazaki H, Imai M, Hirayama T, Saburi S, Tanaka M, Maruyama M, et al Establishment of feeder-independent cloned caprine trophoblast cell line which expresses placental lactogen and interferon tau. Placenta. 2002;23(8–9):613–30. 12361681 10.1053/plac.2002.0846

[17] Ramsoondar J, Christopherson RJ, Guilbert LJ, Wegmann TG. A porcine trophoblast cell line that secretes growth factors which stimulate porcine macrophages. Biol Reprod. 1993;49(4):681–94. 7692989 10.1095/biolreprod49.4.681

[18] Fléchon JE, Laurie S, Notarianni E. Isolation and characterization of a feeder-dependent, porcine trophectoderm cell line obtained from a 9-day blastocyst. Placenta. 1995;16(7):643–58. 8577663 10.1016/0143-4004(95)90033-0

[19] Ka H, Jaeger LA, Johnson GA, Spencer TE, Bazer FW. Keratinocyte growth factor is up-regulated by estrogen in the porcine uterine endometrium and functions in trophectoderm cell proliferation and differentiation. Endocrinology. 2001;142(6):2303–10. 10.1210/endo.142.6.8194 11356676

[20] Talbot NC, Caperna TJ, Edwards JL, Garrett W, Wells KD, Ealy AD. Bovine blastocyst-derived trophectoderm and endoderm cell cultures: interferon tau and transferrin expression as respective in vitro markers. Biol Reprod. 2000;62(2):235–47. 10642558 10.1095/biolreprod62.2.235

[21] Hashizume K, Shimada A, Nakano H, Takahashi T. Bovine trophoblast cell culture systems: a technique to culture bovine trophoblast cells without feeder cells. Methods Mol Med. 2006;121:179–88. 16251743

[22] Bai H, Sakurai T, Kim MS, Muroi Y, Ideta A, Aoyagi Y, et al Involvement of GATA transcription factors in the regulation of endogenous bovine interferon-tau gene transcription. Mol Reprod Dev. 2009;76(12):1143–52. 10.1002/mrd.21082 19598245

[23] Takahashi K, Yamanaka S. Induction of pluripotent stem cells from mouse embryonic and adult fibroblast cultures by defined factors. Cell. 2006;126(4):663–76. Epub 2006/08/15. 10.1016/j.cell.2006.07.024 16904174

[24] Sumer H, Liu J, Malaver-Ortega LF, Lim ML, Khodadadi K, Verma PJ. NANOG is a key factor for induction of pluripotency in bovine adult fibroblasts. J Anim Sci. 2011;89(9):2708–16. 10.2527/jas.2010-3666 21478453

[25] Ezashi T, Telugu BP, Alexenko AP, Sachdev S, Sinha S, Roberts RM. Derivation of induced pluripotent stem cells from pig somatic cells. Proc Natl Acad Sci U S A. 2009;106(27):10993–8. 10.1073/pnas.0905284106 19541600 PMC2698893

[26] Honda A, Hirose M, Hatori M, Matoba S, Miyoshi H, Inoue K, et al Generation of induced pluripotent stem cells in rabbits: potential experimental models for human regenerative medicine. J Biol Chem. 2010;285(41):31362–9. 10.1074/jbc.M110.150540 20670936 PMC2951210

[27] Fujishiro SH, Nakano K, Mizukami Y, Azami T, Arai Y, Matsunari H, et al Generation of naive-like porcine-induced pluripotent stem cells capable of contributing to embryonic and fetal development. Stem Cells Dev. 2013;22(3):473–82. 10.1089/scd.2012.0173 22889279 PMC3549629

[28] Ezashi T, Matsuyama H, Telugu BP, Roberts RM. Generation of colonies of induced trophoblast cells during standard reprogramming of porcine fibroblasts to induced pluripotent stem cells. Biol Reprod. 2011;85(4):779–87. 10.1095/biolreprod.111.092809 21734265 PMC3184293

[29] Chen Y, Wang K, Gong YG, Khoo SK, Leach R. Roles of CDX2 and EOMES in human induced trophoblast progenitor cells. Biochem Biophys Res Commun. 2013;431(2):197–202. 10.1016/j.bbrc.2012.12.135 23313847 PMC3570630

[30] McMahon AP, Bradley A. The Wnt-1 (int-1) proto-oncogene is required for development of a large region of the mouse brain. Cell. 1990;62(6):1073–85. 2205396 10.1016/0092-8674(90)90385-r

[31] Okita K, Ichisaka T, Yamanaka S. Generation of germline-competent induced pluripotent stem cells. Nature. 2007;448(7151):313–7. 10.1038/nature05934 17554338

[32] Yusa K, Zhou L, Li MA, Bradley A, Craig NL. A hyperactive piggyBac transposase for mammalian applications. Proc Natl Acad Sci U S A. 2011;108(4):1531–6. 10.1073/pnas.1008322108 21205896 PMC3029773

[33] Okita K, Nakagawa M, Hyenjong H, Ichisaka T, Yamanaka S. Generation of mouse induced pluripotent stem cells without viral vectors. Science. 2008;322(5903):949–53. 10.1126/science.1164270 18845712

[34] Woltjen K, Michael IP, Mohseni P, Desai R, Mileikovsky M, Hämäläinen R, et al piggyBac transposition reprograms fibroblasts to induced pluripotent stem cells. Nature. 2009;458(7239):766–70. 10.1038/nature07863 19252478 PMC3758996

[35] Tsukiyama T, Asano R, Kawaguchi T, Kim N, Yamada M, Minami N, et al Simple and efficient method for generation of induced pluripotent stem cells using piggyBac transposition of doxycycline-inducible factors and an EOS reporter system. Genes Cells. 2011;16(7):815–25. Epub 2011/06/11. 10.1111/j.1365-2443.2011.01528.x 21658168

[36] Niwa H, Yamamura K, Miyazaki J. Efficient selection for high-expression transfectants with a novel eukaryotic vector. Gene. 1991;108(2):193–9. 1660837 10.1016/0378-1119(91)90434-d

[37] Kawaguchi T, Tsukiyama T, Kimura K, Matsuyama S, Minami N, Yamada M, et al Generation of Naïve Bovine Induced Pluripotent Stem Cells Using PiggyBac Transposition of Doxycycline-Inducible Transcription Factors. PLoS One. 2015;10(8):e0135403 10.1371/journal.pone.0135403 26287611 PMC4544884

[38] Gafni O, Weinberger L, Mansour AA, Manor YS, Chomsky E, Ben-Yosef D, et al Derivation of novel human ground state naive pluripotent stem cells. Nature. 2013;504(7479):282–6. 10.1038/nature12745 24172903

[39] Ezashi T, Das P, Roberts RM. Low O2 tensions and the prevention of differentiation of hES cells. Proc Natl Acad Sci U S A. 2005;102(13):4783–8. 10.1073/pnas.0501283102 15772165 PMC554750

[40] Xu RH, Chen X, Li DS, Li R, Addicks GC, Glennon C, et al BMP4 initiates human embryonic stem cell differentiation to trophoblast. Nat Biotechnol. 2002;20(12):1261–4. 10.1038/nbt761 12426580

[41] Amita M, Adachi K, Alexenko AP, Sinha S, Schust DJ, Schulz LC, et al Complete and unidirectional conversion of human embryonic stem cells to trophoblast by BMP4. Proc Natl Acad Sci U S A. 2013;110(13):E1212–21. 10.1073/pnas.1303094110 23493551 PMC3612666

[42] Kubaczka C, Senner C, Araúzo-Bravo MJ, Sharma N, Kuckenberg P, Becker A, et al Derivation and maintenance of murine trophoblast stem cells under defined conditions. Stem Cell Reports. 2014;2(2):232–42. 10.1016/j.stemcr.2013.12.013 24527396 PMC3923226

[43] Ohinata Y, Tsukiyama T. Establishment of trophoblast stem cells under defined culture conditions in mice. PLoS One. 2014;9(9):e107308 10.1371/journal.pone.0107308 25203285 PMC4159327

[44] Nakano H, Shimada A, Imai K, Takezawa T, Takahashi T, Hashizume K. Bovine trophoblastic cell differentiation on collagen substrata: formation of binucleate cells expressing placental lactogen. Cell Tissue Res. 2002;307(2):225–35. 10.1007/s00441-001-0491-x 11845329

[45] Strumpf D, Mao CA, Yamanaka Y, Ralston A, Chawengsaksophak K, Beck F, et al Cdx2 is required for correct cell fate specification and differentiation of trophectoderm in the mouse blastocyst. Development. 2005;132(9):2093–102. 10.1242/dev.01801 15788452

[46] Chen L, Yabuuchi A, Eminli S, Takeuchi A, Lu CW, Hochedlinger K, et al Cross-regulation of the Nanog and Cdx2 promoters. Cell Res. 2009;19(9):1052–61. 10.1038/cr.2009.79 19564890

[47] Kinoshita M. How are pluripotent cells captured in culture? Reprod Med Biol. 2014. 10.1007/s12522-014-0199-8 PMC4490168 26161037

[48] Berg DK, Smith CS, Pearton DJ, Wells DN, Broadhurst R, Donnison M, et al Trophectoderm lineage determination in cattle. Dev Cell. 2011;20(2):244–55. 10.1016/j.devcel.2011.01.003 21316591

[49] Ushizawa K, Takahashi T, Hosoe M, Ishiwata H, Kaneyama K, Kizaki K, et al Global gene expression analysis and regulation of the principal genes expressed in bovine placenta in relation to the transcription factor AP-2 family. Reprod Biol Endocrinol. 2007;5:17 10.1186/1477-7827-5-17 17462098 PMC1867817

[50] Cauffman G, Van de Velde H, Liebaers I, Van Steirteghem A. Oct-4 mRNA and protein expression during human preimplantation development. Mol Hum Reprod. 2005;11(3):173–81. 10.1093/molehr/gah155 15695770

[51] Kuijk EW, Du Puy L, Van Tol HT, Oei CH, Haagsman HP, Colenbrander B, et al Differences in early lineage segregation between mammals. Dev Dyn. 2008;237(4):918–27. 10.1002/dvdy.21480 18330925

[52] Kobolak J, Kiss K, Polgar Z, Mamo S, Rogel-Gaillard C, Tancos Z, et al Promoter analysis of the rabbit POU5F1 gene and its expression in preimplantation stage embryos. BMC Mol Biol. 2009;10:88 10.1186/1471-2199-10-88 19732419 PMC2751759

[53] Cooke FN, Pennington KA, Yang Q, Ealy AD. Several fibroblast growth factors are expressed during pre-attachment bovine conceptus development and regulate interferon-tau expression from trophectoderm. Reproduction. 2009;137(2):259–69. 10.1530/REP-08-0396 18996977

[54] Fields SD, Hansen PJ, Ealy AD. Fibroblast growth factor requirements for in vitro development of bovine embryos. Theriogenology. 2011;75(8):1466–75. 10.1016/j.theriogenology.2010.12.007 21295834

[55] Ozawa M, Yang QE, Ealy AD. The expression of fibroblast growth factor receptors during early bovine conceptus development and pharmacological analysis of their actions on trophoblast growth in vitro. Reproduction. 2013;145(2):191–201. 10.1530/REP-12-0220 23241344

[56] Bai H, Sakurai T, Someya Y, Konno T, Ideta A, Aoyagi Y, et al Regulation of trophoblast-specific factors by GATA2 and GATA3 in bovine trophoblast CT-1 cells. J Reprod Dev. 2011;57(4):518–25. 21606631 10.1262/jrd.10-186k

