# Supplementary figures and images for: Derivation of Induced Trophoblast Cell Lines in Cattle by Doxycycline-Inducible piggyBac Vectors
Source: PLoS One. 2016 Dec 1;11(12):e0167550. doi: 10.1371/journal.pone.0167550 (PMC5132304; doi:10.1371/journal.pone.0167550)

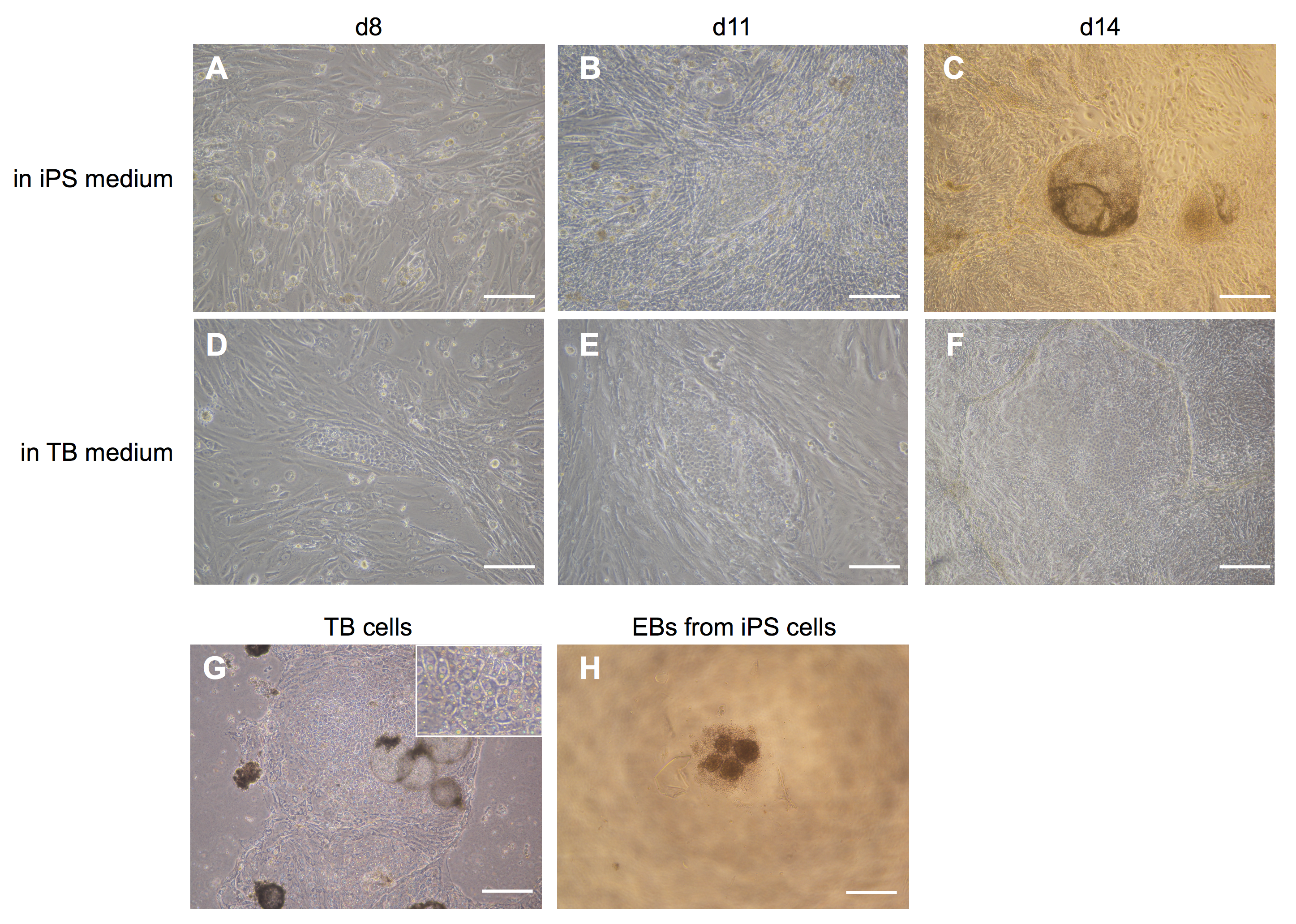

Supplement: S1 Fig — (A)–(C) Appearance of colonies at day 8 (A), day 11 (B), day 14 (C) in iPS medium. (D)–(F) Appearance of colonies at day 8 (D), day 11 (E), day 14 (F) in TB medium. (G) TB cells derivated from CT-1 cells. (H) EBs from iPS cells. (A)–(H), scale bars = 500 μm. (TIF) [file pone.0167550.s001.tif]

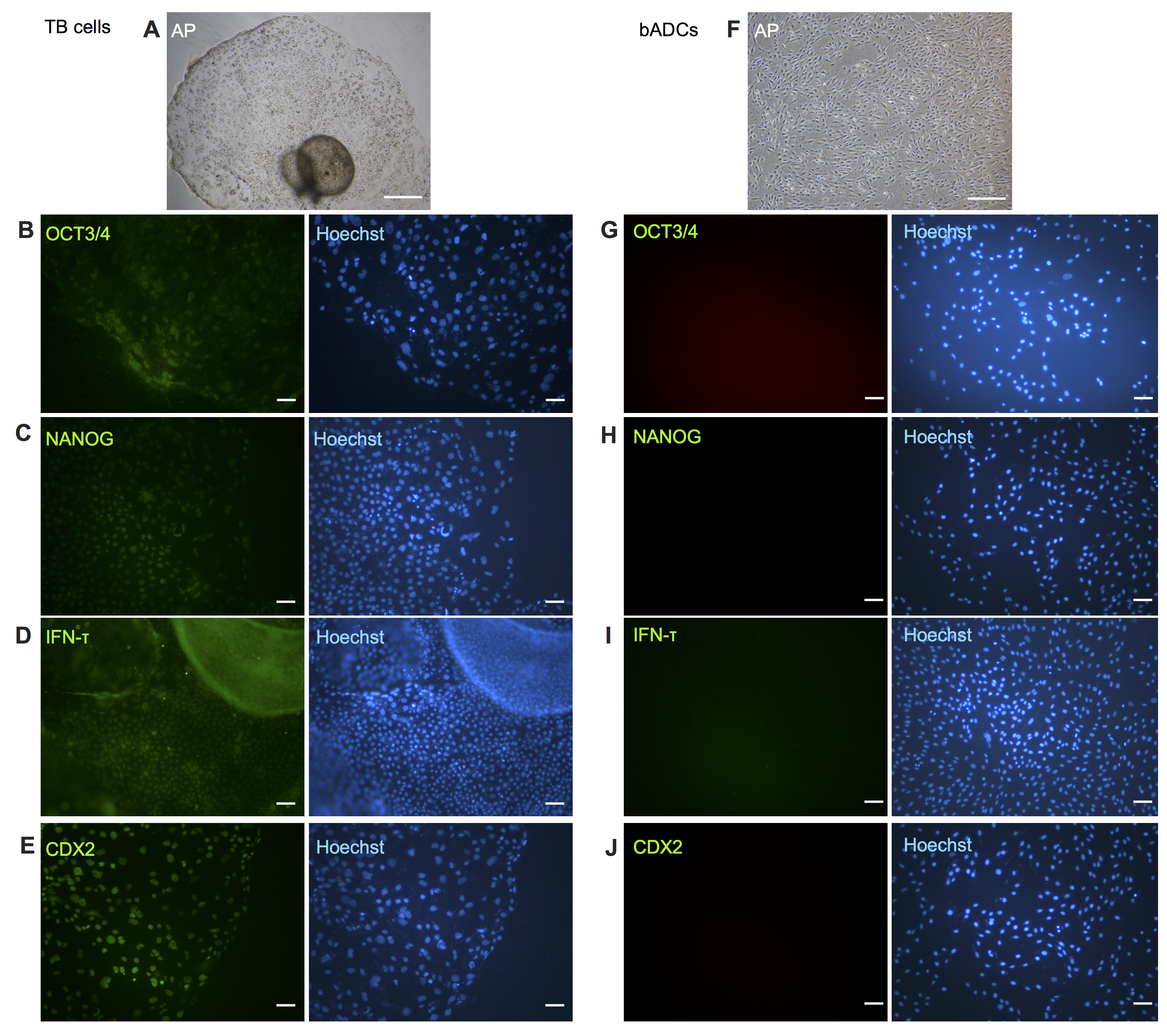

Supplement: S2 Fig — (A) Alkaline phosphatase activity in TBcells. (B) OCT3/4 expression in TB cells. (C) NANOG expression in TB cells. (D) IFN-τ expression in TB cells. (E) CDX2 expression in TB cells. (F) Alkaline phosphatase activity in bADCs. (G) OCT3/4 expression in bADCs. (H) NANOG expression in bADCs. (I) IFN-τ expression in bADCs. (J) CDX2 expression in bADCs. (A), (F) scale bars = 500 μm. (B)–(E), (G)–(J), scale bars = 100 μm. (TIF) [file pone.0167550.s002.tif]

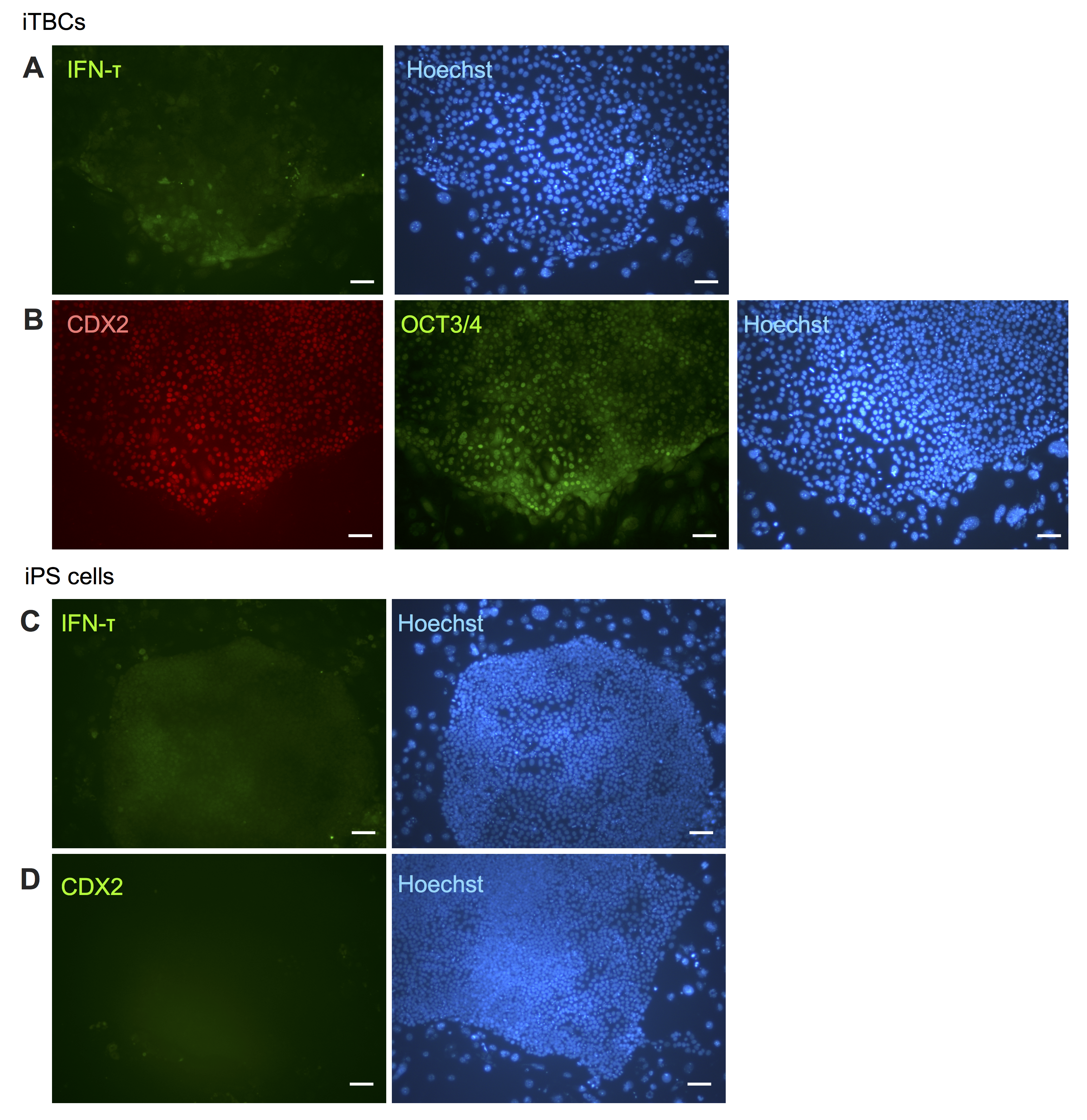

Supplement: S3 Fig — (A) IFN-τ expression in biTBCs. (B) CDX2 (red) and OCT3/4 (green) expression in biTBCs. (C) IFN-τ expression in biPSCs. (D) CDX2 expression in biPSCs. (A)-(D) scale bars = 100 μm. (TIF) [file pone.0167550.s003.tif]
